# 3D‐Printed Scaffolds Promote Enhanced Spinal Organoid Formation for Use in Spinal Cord Injury

**DOI:** 10.1002/adhm.202404817

**Published:** 2025-07-23

**Authors:** Guebum Han, Nicolas S. Lavoie, Nandadevi Patil, Olivia G. Korenfeld, Hyunjun Kim, Manuel Esguerra, Daeha Joung, Michael C. McAlpine, Ann M. Parr

**Affiliations:** ^1^ Department of Mechanical Engineering University of Minnesota Minneapolis MN 55455 USA; ^2^ Department of Neurosurgery Stem Cell Institute University of Minnesota Minneapolis MN 55455 USA; ^3^ Department of Neuroscience University of Minnesota Minneapolis MN 55455 USA; ^4^ Department of Physics Virginia Commonwealth University Richmond VA 23284 USA

**Keywords:** 3D‐printed scaffold, functional recovery, human induced pluripotent stem cells, spinal cord organoids, spinal neural progenitor cells

## Abstract

The transplantation of regionally specific spinal neural progenitor cells (sNPCs) has shown promise for functional restoration after spinal cord injury (SCI) by forming connections with host neural circuits. Here, 3D‐printed organoid scaffolds for transplantation using clinically relevant human induced pluripotent stem cell‐derived regionally specific sNPCs is developed. Scaffolds with microscale channels are printed, and sNPCs are subsequently printed within these channels. The scaffolds direct axonal projections along the channels and guide the cells to simulate in vivo‐like conditions, leading to more effective cell maturation and the development of neuronal networks crucial for restoring function after SCI. The scaffolds, with organoids assembled along their lengths, are transplanted into the transected spinal cords of rats. This significantly promotes the functional recovery of the rats. At 12 weeks post‐transplantation, the majority of the cells in the scaffolds differentiate into neurons and integrate into the host spinal cord tissue. These results demonstrate their potential to create a relay system along the spinal cord and form synapses in both the rostral and caudal directions relative to the scaffold. It is envisioned that combining sNPCs, organoid assembly, and 3D printing strategies can ultimately lead to a transformative treatment approach for SCI.

## Graphical abstract

3D‐printed organoid scaffolds with microscale channels are developed to enhance spinal cord injury recovery by guiding region‐specific spinal neural progenitor cells. These scaffolds promote axonal growth, cell maturation, and neuronal network formation. When transplanted into rat spinal cords, they significantly improve functional recovery, showing promise for treating spinal cord injuries by creating a relay system.

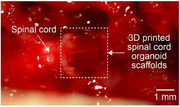

## 1 Introduction

Despite significant public health advancements in clinical management that improve patient quality of life,^[^ ^1^, ^2^, ^3^ ^]^ the occurrence of spinal cord injury (SCI) has remained stable. Within the United States, SCI has affected a significant number of individuals: ≈302 000 persons, with estimates ranging from 255 000 to 383 000 persons.^[^ ^4^ ^]^ Unfortunately, there are no treatments currently available for SCI. Acknowledging the complexity of SCI, any new treatment options would be beneficial.

In accordance with earlier findings,^[^ ^5^, ^6^, ^7^, ^8^, ^9^ ^]^ the transplantation of regionally specific neural progenitor cells is an important approach for functional restoration. Regionally specific spinal neural progenitor cells (sNPCs) formed functional connections with host neural circuits across the spinal cord lesion.^[^ ^6^ ^]^ It is necessary to define populations of transplanted cells and administer regionally specific cells to the damaged area to maximize regenerative capacity. In addition, defining the mechanism of action of these cells is challenging. Many studies on rodents have demonstrated functional benefits of differing therapies,^[^ ^10^, ^11^, ^12^, ^13^, ^14^, ^15^ ^]^ but these have been predominantly neuroprotective mechanisms in acute and subacute injuries that will not translate to chronic SCI. Thus, new strategies should be pursued, such as establishing a relay mechanism by integrating transplanted cells into the neural circuitry. Spinal cord organoids are the ideal substrates for this endeavor, as they are the most anatomically similar to the host spinal cord.

3D bioprinting strategies for neural stem cell transplantation have shown the potential to advance therapies for SCI. For instance, directly injecting cells into a lesion cavity poses the challenge of insufficient structural support.^[^ ^16^ ^]^ 3D‐printed scaffolds have mitigated this issue by offering structural support and providing mechanical and biological guidance for the cells.^[^ ^17^, ^18^, ^19^, ^20^, ^21^, ^22^, ^23^ ^]^ Moreover, 3D printing technologies have demonstrated the ability to create cell‐laden scaffolds that can match the shape of the lesion area,^[^ ^17^, ^24^ ^]^ which could potentially improve graft‐host interactions post‐transplantation.^[^ ^6^ ^]^ However, despite these advantages, the application of 3D‐printed scaffold strategies on multiple organ‐specific cell types, such as organoids, remains nascent.

To advance the development of regionally specific neural progenitor cells for clinical usage, we created 3D‐printed spinal cord organoid scaffolds using clinically relevant human induced pluripotent stem cell (iPSC)‐derived sNPCs, which have the potential to avoid immune rejection.^[^ ^25^ ^]^ Previous studies, including our own, have demonstrated that the iPSC‐derived regionally specific sNPCs maintain their regional specificity after transplantation.^[^ ^5^, ^6^, ^25^ ^]^ The majority of sNPCs differentiated into neurons and oligodendrocytes and expressed ventral cervical/thoracic HOX genes.^[^ ^6^, ^7^ ^]^ These cells showed promise in differentiating in vivo to replace lost or damaged cells, thereby replicating spinal cord tissue.^[^ ^6^, ^26^ ^]^ Although there are a variety of natural and synthetic polymers that can be used for printing scaffolds,^[^ ^27^, ^28^ ^]^ no single polymer exhibits all of the required properties optimally.^[^ ^29^ ^]^ Silicone is a widely recognized material used in medical applications due to its high biocompatibility, excellent oxidation resistance, and high gas permeability, which supports the survival of oxygen‐demanding cells.^[^ ^30^ ^]^ Our lab had previously established a comprehensive in vitro characterization of silicone scaffolds.^[^ ^16^, ^30^ ^]^ Furthermore, the non‐degradable nature of silicone makes it a suitable scaffold material for studying the development of printed cells into organoids under mechanical guidance without the confounding influence of scaffold degradation. Given these advantages, we developed 3D bioprinted spinal cord organoid silicone scaffolds in vivo to promote functional recovery in a rat model with a transected spinal cord. An extrusion‐based multi‐material printing system placed these sNPCs into microchannels within 3D‐printed scaffolds, which were created on sacrificial layers to avoid damage during detachment from the culture plate before transplantation. Following in vitro culture, the 3D‐printed sNPC‐laden scaffolds matured into spinal cord organoid scaffolds. Subsequently, these organoid scaffolds were transplanted into rats to re‐establish spinal cord circuitry across the lesion site. To understand their formation process and efficacy, we examined neuronal maturity, neural network formation, and organoid assembly in vitro, as well as their ability to integrate into the host spinal cord post‐transplantation in vivo.

## 2 Results

### 2.1 Printing and Transplantation Processes of 3D Spinal Cord Organoid Scaffolds

**Figure 1:**
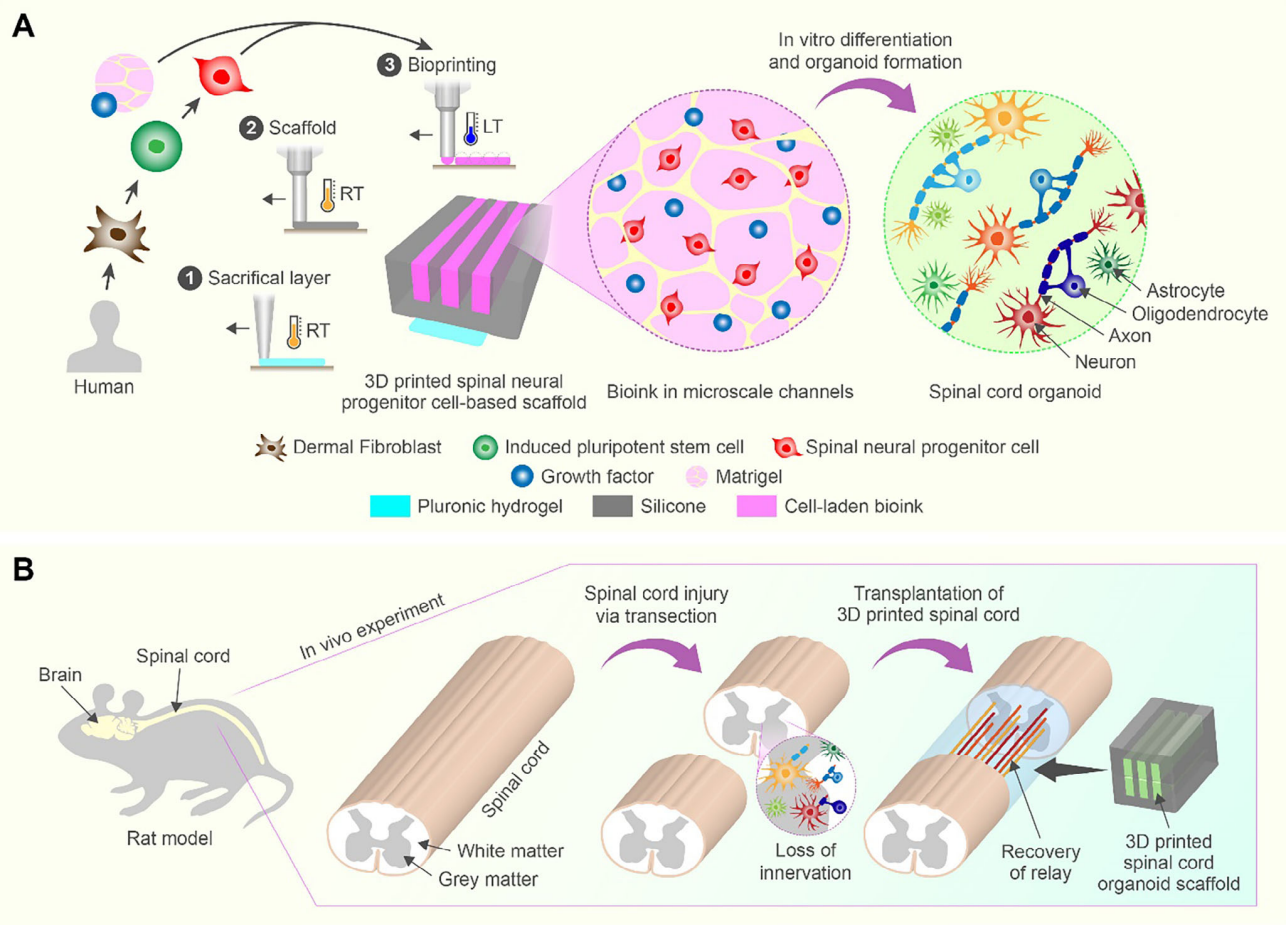
Schematic of experiments. A) Printing process of spinal cord organoid scaffolds. The multi‐head extrusion 3D printer created a Pluronic sacrificial layer on a glass substrate and constructed a silicon scaffold with three microscale channels in a layer‐by‐layer manner. The cell‐laden ink, containing sNPCs, Matrigel, and neural media with growth factors, was positioned within the channels via point dispensing. The Pluronic sacrificial layer and silicone scaffold were printed at room temperature (RT), while the bioink was printed at a low temperature (LT) of 4 °C. The 3D‐printed spinal cord scaffold was cultured for in vitro differentiation until organoid formation. B) Transplantation of 3D‐printed spinal cord organoid scaffolds. The sacrificial layer was dissolved during culture. The 3D‐printed spinal cord organoid scaffolds were detached from the glass substrates by cutting the edges where they made contact. Two of these scaffolds were assembled and implanted into the 1.8 mm gap in the spinal cord of a rat, created by the transection injury, to immediately promote functional recovery.

**Figure** **1** shows a schematic overview of the printing and transplantation processes of 3D‐printed spinal cord organoid scaffolds into a rat model of transection injury. The Experimental Section provides detailed information about the materials and printing conditions used to create the scaffolds. An extrusion‐based multi‐material printing system was used to print a Pluronic hydrogel sacrificial layer on a glass substrate (Figure 1A). Subsequently, a silicone scaffold was printed on top of this layer, with its edges in contact with the glass substrate. The sacrificial layer was designed to dissolve in cell media during culture, while the remaining silicone edges were intended to prevent the scaffold from floating in cell media and to facilitate its removal from the glass substrate for transplantation by cutting the edges carefully to avoid damage. The silicone scaffolds for transplantation had dimensions of ≈1.6 mm in width, 0.65 mm in height, and 2 mm in length. For the in vitro study, the length was ≈5 mm, and the sacrificial layer and the silicone contact edges were excluded (Figure S1, Supporting Information), as there was no need to remove the scaffold from the substrate. Each scaffold contained three channels with dimensions of ≈200 µm in width and 440 µm in height.

The open scaffold design and Matrigel‐based cell‐laden ink were based on our previous study.^[^ ^30^ ^]^ Specifically, open channels were chosen because they facilitated imaging, enabled convenient contamination monitoring during long‐term culture, shortened the diffusion distance for nutrients and oxygen, and allowed quick assessment of cell‐laden channel integrity before transplantation. During transplantation, this stacked design protected the cells and provided a structure mimicking the grey matter in the spinal cord. After curing the silicone scaffolds for at least 5 h, a cell‐laden ink, consisting of sNPCs, Matrigel, and neural media with growth factors, was printed into the three channels. Movie S1 (Supporting Information) shows the printing process of the spinal cord scaffold for transplantation. For visualization purposes, red dyes were added to the cell‐laden ink. The dispensing volume of cell‐laden ink was estimated based on the dimensions of the channels. The printing nozzle tip was maintained at 4 °C using a cooling system, ensuring the printability of the cell‐laden ink. A 10 min post‐printing wait period followed to facilitate the secure adhesion of the ink onto the scaffold channels. The scaffolds were cultured in vitro in cell media. During this stage, the Pluronic sacrificial layer was dissolved. After culturing until the formation of 3D‐printed spinal cord organoid scaffolds, based on the in vitro study, they were separated from the glass substrates by cutting along the silicone edges in contact. Two scaffolds were assembled and implanted within the 1.8 mm gap of the 2 mm‐transected spinal cord of a rat immediately after the cut (Figure 1B). The results of the in vitro study and the in vivo transplantation of the 3D‐printed spinal cord organoid scaffolds are presented and discussed in the following sections.

### 2.2 3D Bioprinted Human iPSC‐Derived sNPCs Differentiated into Multiple Organ‐Specific Cell Types and Maintained Neuronal Identity for at Least One Year In Vitro

**Figure 2:**
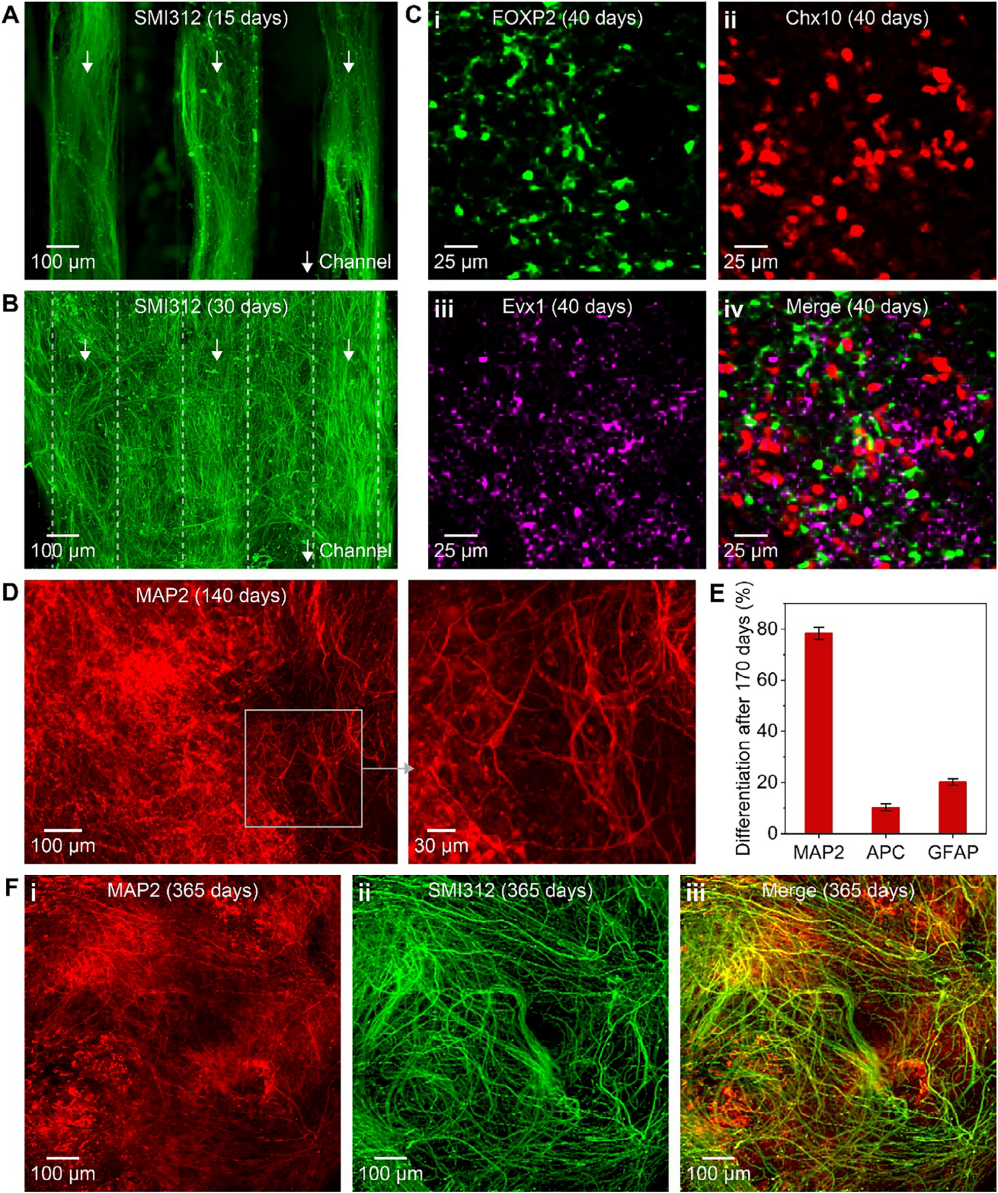
IHC results of the 3D‐printed spinal cord scaffold in vitro. A) Expression of SMI312 showing axonal and dendritic projections guided by the 3D‐printed channels at 15 days. B) Expression of SMI312 showing the extension of axonal and dendritic projections over the top of the scaffold channel at 30 days. The arrows with the dashed lines indicate the scaffold channels beneath the neuronal outgrowth network. C) Expression of i) FOXP2, ii) Chx10, and iii) Evx1 representing V1, V2a, and V0 neurons, respectively, in the scaffold at 40 days. iv) shows the merged image. D) Expression of MAP2 showing neurons that maintained their growth along the scaffold at 140 days. The boxed region is magnified. E) Quantification of the percentage of cells expressing the markers MAP2, APC, and GFAP in the scaffold at 170 days. The data represent mean ± standard error. F) Expression of i) MAP2 and ii) SMI312 showing 3D‐printed spinal cord scaffolds survived for at least 365 days post‐printing and maintained their neuronal identity. iii) shows the merged image.

The fate of human iPSC‐derived sNPCs that were printed in the scaffold channels was analyzed in vitro at several time points, including 15, 30, 40, 140, 170, and 365 days after printing. **Figure** **2** shows representative images of printed cells labeled with SMI312, Evx1, FOXP2, Chx10, MAP2, GFAP, and APC. Immunohistochemistry (IHC) was conducted to examine these labeled cells.

Figure 2A shows 3D bioprinted sNPCs in scaffold channels after 15 days of culture, labeled with the mature neuronal marker SMI312. This indicates axonal growth guided by the microscale channels during the initial phase. Figure 2B, taken from the top, viewing the open surface of the scaffold, shows axons filling the scaffold channels (Figure 2A) and extending to cover the top, forming a neuronal network after 30 days of culture. Figure 2C shows the bioprinted sNPCs expressing Chx10, FOXP2, and Evx1 after 40 days of culture, representing V2a, V1, and V0 neurons, respectively. This demonstrated their differentiation into multiple organ‐specific cell types and supported the concept of organoid formation. Further, using confocal microscopy, we observed “layering” of these markers similar to laminae in the spinal cord. Figure 2D and Figure S2 (Supporting Information) show the expression of MAP2 in the sNPCs after 140 days of culture, indicating the survival of axons and dendrites, as well as the maturation and maintenance of neurons along the scaffold. The quantitative analysis in Figure 2E, obtained after 170 days of culture, revealed that a majority of the cells expressed MAP2 (78.35 ± 2.37%), whereas a minority of cells expressed markers of astrocytes (GFAP: 20.18 ± 1.25%) and mature oligodendrocytes (APC: 10.20 ± 1.40%). Figure 2F presents the expression of SMI312 and MAP2 in the tissue construct assembled from the 3D‐printed sNPC‐laden scaffold after 365 days of culture. This demonstrated that the tissue exhibited long‐term survival, maintained its neuronal identity, and showed highly organized bundles of axons.

### 2.3 Transcriptional Profiling of 3D Bioprinted iPSC‐Derived sNPCs Supports the Generation of Mature Neuronal Identity and Organoids In Vitro

**Figure 3:**
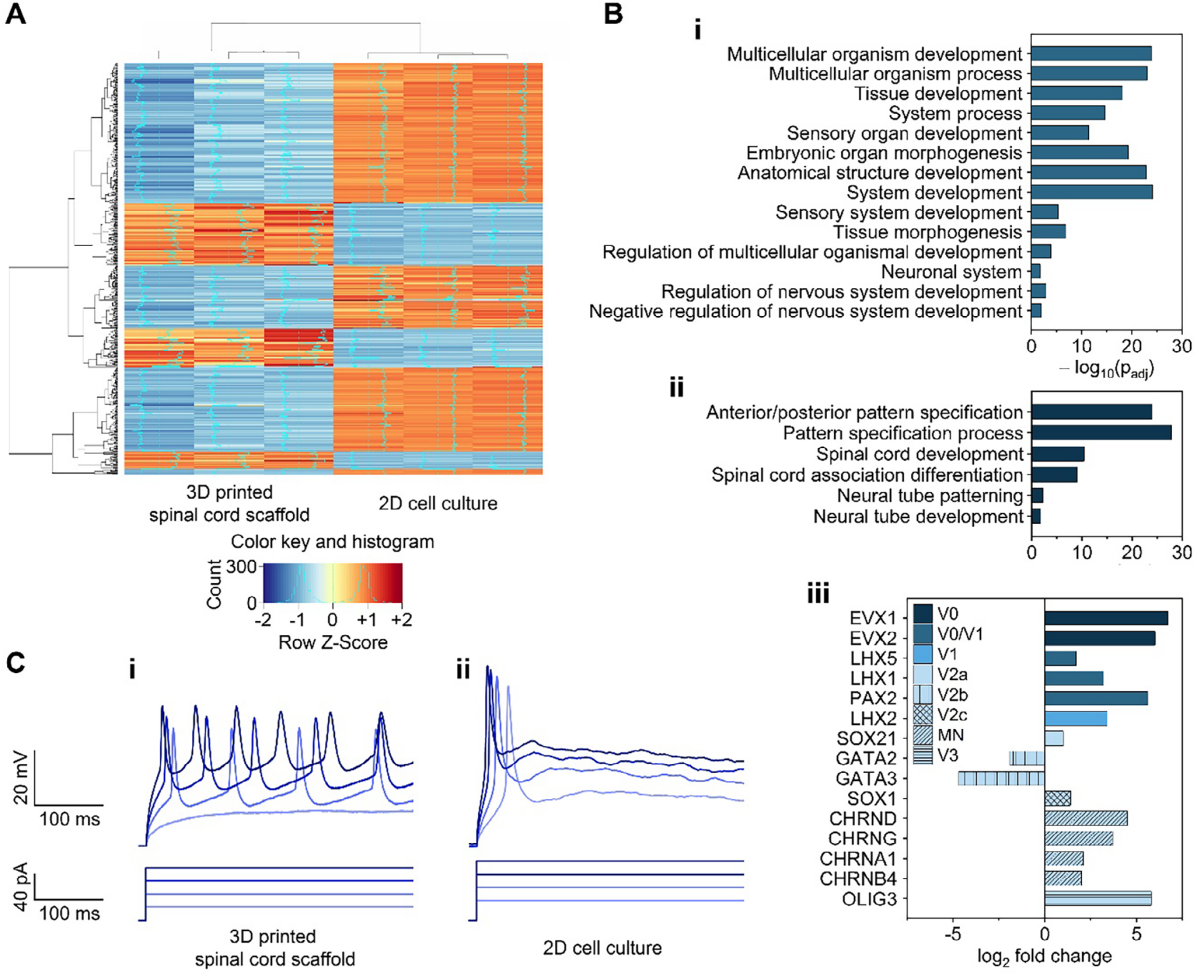
Results of RNA seq and electrophysiology for the 3D‐printed spinal cord scaffold in vitro. A) Hierarchical clustering shows a distinct separation between the 3D‐printed spinal cord scaffold and 2D cell culture after 25 days post‐differentiation from iPSCs (equivalent to 21 days of culture after printing). Read counts were normalized and log‐transformed. The first three and last three columns represent sample replicates. Blue indicates lower expression levels, while red indicates higher expression levels. The color key displays the intensity associated with the normalized expression values. This displays similarity in expression between samples in the top 500 differentially expressed genes and reveals that HOXB4, HOXA5, HOXB7, and HOXB9 are among the most differentially expressed genes. This further suggests that organoids maintain a spinal cord positional identity. B) Gene ontology analysis reveals enrichment of i) spinal cord organogenesis and ii) regionalization in 3D‐printed sNPCs compared to their 2D cultured equivalents. 3D‐printed sNPCs express iii) heterogenous post‐mitotic ventral spinal cord neuronal markers, while 2D cultured cells are primarily fated to V2b interneurons. C) Patch clamp electrophysiological recordings on single cells show that i) sNPCs printed within the scaffold channels exhibit enhanced maturity compared with ii) 2D cultured cells. The recordings were performed 19 days after differentiation from iPSCs (equivalent to 15 days of culture after printing).

RNA sequencing (RNA‐seq) was performed to elucidate differential gene expression patterns in a 3D‐printed sNPC‐laden scaffold model versus 2D cell culture after 25 days post‐differentiation from iPSCs. When counted from the printing time point for the scaffolds, the 25 days are equivalent to 21 days of culture after printing. Unsupervised hierarchical clustering (**Figure** **3**A) demonstrated a recognizable separation between the two groups with minimal variability within groups. The cluster analysis of the differentially expressed genes (DEGs) showed marked differences in gene expression levels between 3D‐printed spinal cord organoid scaffolds and 2D cell culture. Further analysis identified 500 DEGs, revealing HOXB4, HOXA5, HOXB7, and HOXB9 as the most common DEGs. According to the functional annotation in the Gene Ontology (GO) database, the upregulated DEGs were primarily associated with organoid formation (Figure 3B‐i), regionalization (Figure 3B‐ii), and post‐mitotic neuronal formation (Figure 3B‐iii), consistent with the IHC results (Figure 2). These findings suggested that cells within the 3D bioprinted constructs exhibited an organoid‐like phenotype with diverse, spinal cord‐specific neuronal identities compared to those in 2D culture conditions.

### 2.4 3D Bioprinted iPSC‐Derived sNPCs Produced Functionally Active Neurons In Vitro

Patch clamp electrophysiology was conducted to investigate the electrical properties of a 3D‐printed sNPC‐laden scaffold model compared to sNPCs in 2D cell culture, 19 days after differentiation from iPSCs (Figure S3, Supporting Information). When measured from the time of printing, the 19‐day period corresponds to 15 days of culture following printing. Whole‐cell patch clamp electrophysiology was used to analyze current‐induced action potentials (Figure 3C). Inward sodium and delayed rectifying potassium currents indicated the presence of voltage‐gated sodium and potassium channels on their membranes and a functional neuronal profile. All recorded 3D‐printed sNPC‐laden scaffold models generated continuous repetitive spikes (Figure 3C‐i), unlike those in the 2D culture (Figure 3C‐ii). These results demonstrated that the sNPCs in the 3D‐printed scaffold channels differentiated into functional neurons and exhibited enhanced maturity compared with those in 2D culture 15 days after printing.

### 2.5 Transplantation of 3D Bioprinted Spinal Cord Organoid Scaffolds Improves Functional Recovery of Rats with SCI

**Figure 4:**
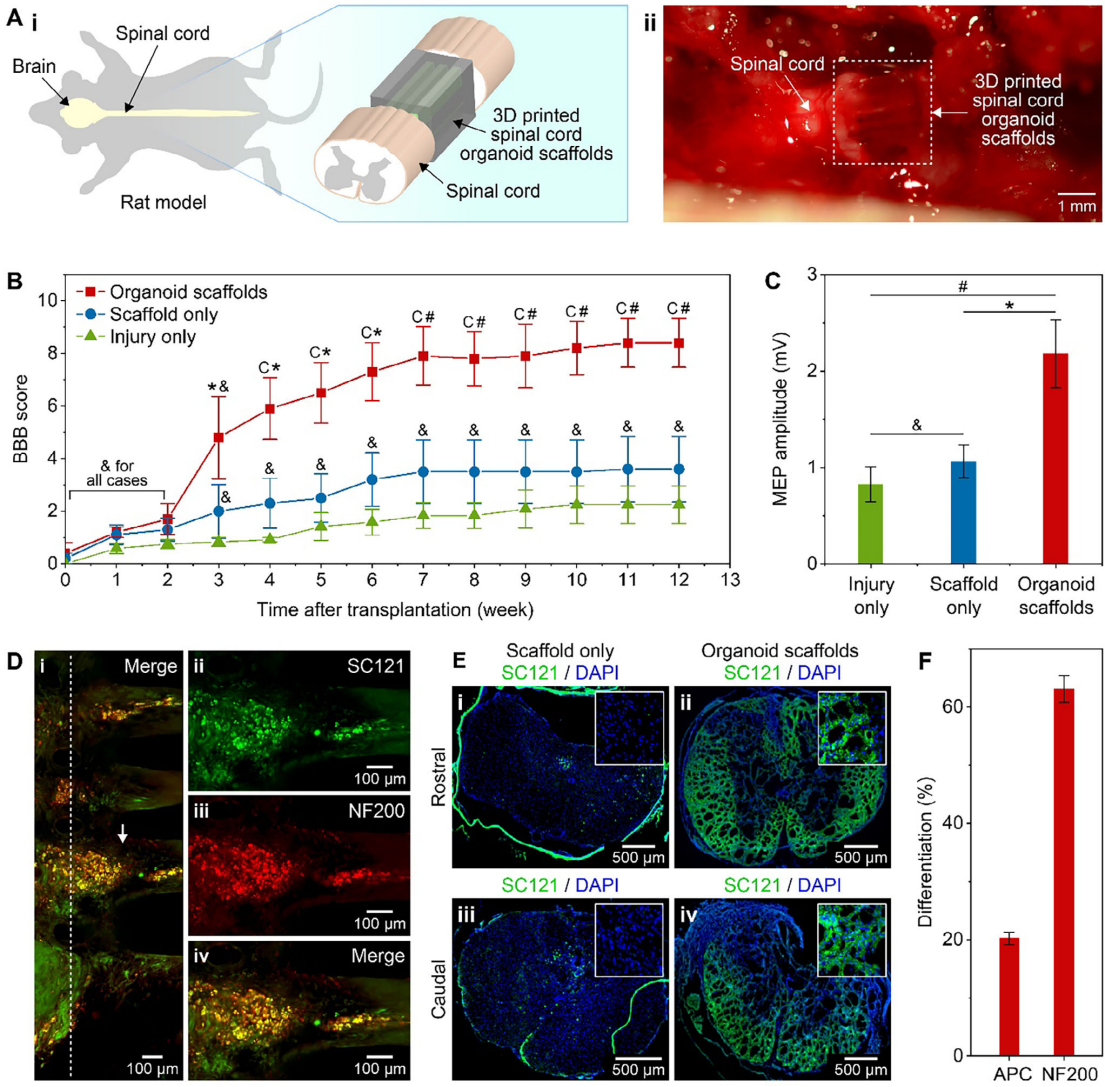
Functional recovery of transected rats after transplantation of the 3D‐printed organoid scaffolds. A) i) Schematic and ii) image showing the transplantation of the 3D‐printed organoid scaffolds into the transected spinal cords of rats. B) BBB scores showing a significant increase from week 3 to 12 in the 3D‐printed organoid group compared to the injury‐only and scaffold‐only groups. Repeated measures ANOVA were used to assess the BBB scores. Significance levels were set as ^*^ *p*< 0.05, *p*< 0.01, ^c^ *p*< 0.001, and ^&^ *p* for non‐significance. The data are presented as mean ± standard error of the mean. C) MEPs were measured in the injury‐only, scaffold‐only, and 3D‐printed organoid scaffold groups. The organoid scaffold group exhibited higher MEP amplitudes compared to the injury‐only and scaffold‐only groups. A one‐way ANOVA with the Bonferroni post hoc test was used to evaluate the MEP results. Significance levels were set as ^*^ *p*< 0.05, *p*< 0.01, and ^&^ *p* for non‐significance. The data are presented as mean ± standard error of the mean. D) Co‐localization of SC121 (green) and NF200 (red) in the scaffold 12 weeks post‐transplantation. i) The left side of the white dashed line represents the direction of the host spinal cord tissue, while the right side represents the direction of the scaffold. The co‐localization showed that the implanted cells and spinal cord tissues were integrated in the vicinity of their bridging area. ii–iv) Higher magnification images of the area indicated by the white arrow in (i). E) Spinal cord sections from the (i and iii) scaffold‐only and (ii and iv) organoid scaffold groups, showing SC121 (green) and DAPI (blue) 12 weeks post‐transplantation. The top and bottom rows represent sections rostral to the lesion site and caudal to the lesion cavity, respectively. F) Quantification of the percent co‐localization of SC121^+^ human cells with APC and NF200 in the rostral and caudal sections of the spinal cord. The data are presented as mean ± standard error of the mean.

The combination of the in vitro results at different time points (Figures 2 and 3) indicated that 3D bioprinted sNPCs in scaffold channels assembled organoids with multiple organ‐specific cell types 40 days after printing. Therefore, before transplantation, 3D‐printed sNPC‐laden scaffolds were cultured for 40 days to become 3D‐printed organoid scaffolds. The scaffolds were then detached from their glass substrates by cutting along their contact edges. Two scaffolds were utilized for each rat. They were assembled with the channel surfaces together, such that one scaffold was positioned ventrally and the other dorsally. These scaffolds were implanted into the 1.8 mm gap created by the 2 mm transection of the spinal cord of a rat immediately following the injury (Figures 1B and 4A; Figure S4, Supporting Information).

After the transplantation, the Basso, Beattie, and Bresnahan (BBB) locomotor assessment was used to evaluate the functional recovery of rats facilitated by the 3D‐printed spinal cord organoid scaffolds. The group with the 3D‐printed organoid scaffolds was compared to the groups with injury only and with empty scaffolds only. Two weeks post‐transplantation, BBB scores for all groups were < 2 (**Figure** **4**B). The BBB scores of the 3D‐printed organoid scaffold group showed a noticeable increase from week 3 (4.8 ± 1.56) to week 7 (7.9 ± 1.11). At the end of 12 weeks post‐transplantation, the 3D‐printed organoid scaffold group exhibited significant functional recovery (8.4 ± 0.93), compared to the groups with scaffolds only (3.6 ± 1.25) and injury only (2.25 ± 0.72). The difference between the injury‐only and scaffold‐only groups was not significant. These results showed that the organoid scaffolds improved the functional recovery of the rats after SCI.

In addition, motor evoked potentials (MEPs) were measured for all groups 12 weeks post‐transplantation. MEPs were elicited by stimulating the motor cortex of the rat brain with a magnetic stimulator and recorded from the gastrocnemius muscle of the rat leg using an inserted needle. The group implanted with the 3D‐printed spinal cord organoid scaffolds (2.18 ± 0.35 mV) showed significantly higher MEP amplitudes than both the empty scaffold‐only group (1.07 ± 0.17 mV) and the injury‐only group (0.83 ± 0.18 mV) (Figure 4C). These findings suggest that the organoid scaffolds enhanced neural connectivity and functional recovery across the transection site, enabling improved transmission of motor signals from the brain through the spinal cord to the muscles compared to the other groups.

### 2.6 Transplantation of 3D Bioprinted Organoid Scaffolds Induced Axonal and Dendritic Projections Across the Lesion Site In Vivo

At 12 weeks post‐transplantation, the scaffolds and adjacent spinal cord tissues were removed from the organoid treatment group. They were tissue‐cleared and then immunoassayed with the human cytoplasmic marker SC121 and the neuronal marker NF200 to identify the axonal projections from the scaffolds (Figure S5, Supporting Information). The co‐localization, as shown in Figure 4D‐i, demonstrated the integration of the implanted cells and host spinal cord tissues near the bridging areas, highlighted by the white dashed line. Additionally, it depicted axonal projections along the channels of the 3D‐printed scaffolds, shown on the right side of the white dashed line. Figure 4D‐ii, iii, and iv show higher magnification images of the channel highlighted by the white arrow in Figure 4D‐i. Further, we observed that these SC121 positive projections form active synaptic networks within and at the interface of the scaffolds and the cut ends of the spinal cord, indicating functional integration of the graft with host neural populations (Figure S6, Supporting Information).

In addition, axial slices of the host spinal cord from the 3D‐printed organoid scaffold group were investigated using IHC. We examined SC121 positive projections (Figure 4E) that were co‐localized either with neuronal marker NF200 or oligodendrocyte marker APC in both rostral and caudal directions to the scaffold (Figure S6, Supporting Information). Figure 4F shows that a majority of the SC121 positive cells differentiated into neurons (63.10 ± 2.31%), while a minority differentiated into oligodendrocytes (20.21 ± 1.07%) in the 3D‐printed organoid scaffold group. However, as expected, no SC121 was observed in either the injury‐only or scaffold‐only groups.

## 3 Discussion

We developed 3D bioprinted spinal cord organoid scaffolds for transplantation into sites of SCI to bridge the post‐injury microenvironment and potentially promote a functional neural relay across the injury site. The 3D scaffolds featuring microscale channels were printed, and clinically relevant human iPSC‐derived regionally specific sNPCs were printed within the channels. The mechanical guidance provided by the scaffolds directed the axonal projections along the channels and promoted cell maturation. After culture, the sNPCs in the scaffold channels developed into organoids along their length. These spinal cord organoid scaffolds enabled functional recovery across the transection injury in rats. These findings suggest that our scaffold approach can restore effective innervation across the lesioned area, relying on the precise placement of cells within it to recapitulate the lost spinal cytoarchitecture.^[^ ^30^, ^31^ ^]^ While the use of scaffolds in SCI is not novel,^[^ ^17^, ^32^, ^33^ ^]^ it remains novel in humans. There are currently no clinical trials listed on ClinicalTrials.gov using a combination of scaffolds and neural cells. Despite optimistic preliminary results obtained by the implantable scaffolds (INSPIRE; ClinicalTrials.gov, NCT02138110), the trial ultimately failed due to the inability to achieve the primary outcome.^[^ ^34^ ^]^ Therefore, more work is needed in combining structure (scaffolds) with substrate (cells). Our approach offers a distinct advancement in that it combines a 3D bioprinting method with sNPCs to guide the formation of organoids with multiple spinal cell types along a scaffold designed for integration and functional recovery at the injury site. This advancement may ultimately improve cell‐to‐cell contact, which is crucial for successful graft‐host interactions.^[^ ^35^ ^]^

3D bioprinted sNPCs in scaffolds developed more mature and regionally specialized neuronal networks compared to 2D in vitro cultures. The majority of the cells exhibited SMI312 and MAP2 positive axonal and dendritic extensions along the scaffold channels in vitro, respectively, indicating the development of guided neuronal networks and neuronal maturity. Similar observations were made in our previous experiments and by others.^[^ ^36^, ^37^, ^38^ ^]^ Furthermore, the networks survived and maintained their identity for at least one year. These cells also expressed V0, V1, and V2a interneurons, which regulate locomotor activity. V2a spinal interneurons, in particular, are essential for motor control by providing excitatory input to motoneurons.^[^ ^39^ ^]^ These findings highlighted the need for regional specificity^[^ ^5^, ^7^, ^40^ ^]^ in transplanted cells to allow functional restoration after SCI. The results of the RNA‐seq and patch clamp electrophysiology supported the hypothesis that sNPCs in 3D‐printed scaffold channels differentiated into functional neurons with greater maturity and improved regionalization compared to those in 2D culture.

The 3D‐printed spinal cord tissue constructs met the definition of an organoid as described by Sloan et al.,^[^ ^41^ ^]^ capturing human‐specific cellular diversity and 3D tissue architecture in a more customizable format. In contrast to our previous cell transplantation study without scaffolds,^[^ ^6^ ^]^ which revealed only two distinct cell populations and no evidence of organoid formation, the RNA‐seq results from the 3D‐printed scaffold group showed significant upregulation of DEGs associated with organoid formation, regionalization, and post‐mitotic neuronal development. Additionally, while 2D cultured cells committed to V1 and V2b lineages, the 3D‐printed scaffold group expressed post‐mitotic factors associated with the ventral layers of the spinal cord after 21 days of culture. As discussed earlier, the 3D‐printed scaffold group also demonstrated in vivo‐like cell maturation, regionalized neuronal network development, and functional neurons compared to 2D culture conditions. These findings suggest that 3D scaffold structures provided a more conducive environment for cells to form organoids with diverse, spinal cord‐specific neuronal identities, potentially offering a platform that better mimics the natural microenvironment of spinal cord tissue in vitro. Furthermore, channels within the scaffolds do provide directionality during the initial phase of seeding the cells into the scaffolds. These cells often migrate along the direction of the channels, leading to a more organized or directional distribution of cells within the scaffold.

The in vivo study, including BBB assessment, MEPs, and IHC, demonstrated that the organoid scaffolds with spinal cord‐specific cell types promoted functional recovery in rats with transection injuries via integration into the host tissue. The functional improvement in the rats that were implanted with 3D‐printed organoids may be due to harmonious integration of the multiple neural pathways, including recruitment of novel interneurons^[^ ^42^ ^]^ and expression of V1, V2a, and V0 interneurons that are regionally specific to the spinal cord relay across the injury site, and therefore, establish a beneficial neural network. Although stacking two organoid scaffolds during implantation might introduce stress to the neuronal networks, the findings of cell survival and functional recovery suggested that this stress was minimal. The expression of synaptophysin throughout the scaffold indicates an integrated, organized neural network characteristic of organoid formation. Further, the host graft synapses at the interface between the 3D‐printed scaffold and cut ends of the spinal cord might be one of the many factors contributing to the functional improvement.^[^ ^43^ ^]^ However, the effect of the direction of neural network formation on the functional recovery was not investigated in this study.

Other studies^[^ ^33^, ^44^ ^]^ have shown functional improvement with higher BBB scores than those found in our study. Those studies opted for different SCI models implanted with 3D‐printed scaffolds loaded with neural cells. Teng et al.^[^ ^44^ ^]^ used a hemisection model of SCI, whereas we have a transection model of SCI. A hemisection model can have local sprouting to the contralateral side, and there is no evidence of cross‐lesional axonal extension.

Most implanted cells differentiated into neurons and populated both the scaffold channels and the junction between the scaffolds and the host tissue with axonal extension, and the connections were predominantly located in the white matter of the spinal cord sections, both rostral and caudal to the organoid scaffold implants. These observations were consistent with the study by Koffler et al.,^[^ ^17^ ^]^ which used 3D‐printed scaffolds with non‐specific neural progenitor cells. These results suggest the potential for a functional relay system along the spinal cord in both rostral and caudal directions from the scaffold implant and the formation/strengthening of synapses between graft‐derived human neurons and host rat neurons.

## 4 Conclusion

3D spinal cord organoid scaffolds were created by combining a multi‐material 3D printing strategy with human‐induced iPSC‐derived regionally specific sNPCs. A 3D scaffold with microscale channels was printed layer‐by‐layer, with sNPCs placed in the channels using a point dispensing method. The 3D‐printed scaffolds influenced the differentiation pattern of sNPCs and provided directional guidance for the cells. The 3D spinal cord organoid scaffolds, which have spinal cord‐specific neuronal identities, promoted a functional neural relay across the injury site in a rat model of complete thoracic transection. Most of the cells within the scaffolds differentiated into neurons, and many extended out of the scaffolds into the host spinal cord in both rostral and caudal directions, forming connections between the graft‐derived human neurons and the host rat neurons.

Future research will focus on the following areas to advance the foundation established by the current study. i) The importance of regional specification along the dorsal‐ventral axis for cell transplantation has been well demonstrated to enhance integration and functional recovery.^[^ ^45^ ^]^ Most of this work has focused on the specification of motor neuron and ventral interneuron populations, as we have also shown in the present study.^[^ ^6^, ^46^, ^47^ ^]^ However, without proper recovery of the complex sensory circuits that in turn modulate motor output, meaningful recovery may not be attainable. To address this, our group and a handful of others have developed and characterized populations of hiPSC‐derived dorsal neurons for use in SCI.^[^ ^7^, ^48^ ^]^ In the future, these dorsal and ventral neural progenitor cells can be used in combination to restore sensorimotor connectivity above and below the site of injury. ii) 3D printing strategies will be developed for the fully automated fabrication of scaffolds that mimic ventral and dorsal tissue regions through the differentiation of precisely positioned, region‐specific sNPCs. iii) Biodegradable scaffolds composed of polycaprolactone (PCL) and poly(lactic‐co‐glycolic acid) (PLGA) will be designed to provide mechanical cues that direct axonal projections and facilitate organoid formation in the desired configuration, while gradually degrading after transplantation. iv) 3D‐printed biodegradable scaffolds will be integrated with region‐specific, clinically relevant sNPC‐laden decellularized extracellular matrix (dECM). We further envision that combining 3D printing technologies with these clinically relevant components (sNPCs and organoid biology) will enable the development of 3D tissue constructs, advancing therapeutic strategies for restoring functional neural relays after SCI.

## 5 Experimental Section

### In Vitro Study Design

Human iPSC‐derived sNPCs that were regionally specific to the human spinal cord were utilized.^[^ ^7^, ^8^ ^]^ In the first part of these in vitro experiments, continuous organoid development along the x and y axes, with dimensions up to ≈5 mm in length and ≈1.6 mm in width, was spatially defined by bioprinting sNPCs into the scaffold channels. The scaffold width was determined based on the size of the rat spinal cord. The cell‐laden scaffolds were examined at different time points of in vitro culture by conducting IHC after tissue clearing, patch clamp electrophysiology, and RNA‐seq analysis.

### In Vitro Cell Culture

iPSCs were differentiated into sNPCs according to the previously published protocol.^[^ ^49^, ^50^ ^]^ Human vitronectin (AF‐140‐09, PeproTech, NJ) was briefly used to passage iPSC cultures, and the cell cultures were maintained at 37°C with 5% CO_2_. To induce differentiation, neuromesoderm progenitor differentiation medium was added to the cell cultures for 24 h. This medium consists of Essential 6 Medium (A1516401, Thermo Fisher Scientific, MA), LDN‐193189 (500 nM, S7507, Selleck Chemicals, TX), and BGJ398 (100 nM, S2183, Selleck Chemicals, TX). On day 2, the cells were placed in accelerated spinal neuroepithelium differentiation medium for 24 h, consisting of Essential 6 Medium supplemented with A83‐01 (500 nM, 2939, Tocris Bioscience, MN), CHIR 99021(4 mM, 4423, Tocris Bioscience, MN), and FGF‐Basic TS (20 ng mL^−1^, HZ‐1291, HumanZyme, IL). On day 3, the cells were placed in accelerated spinal neuroepithelium differentiation medium for 24 h, consisting of Essential 6 Medium supplemented with BGJ398 (100 nM, S2183, Selleck Chemicals, TX), smoothened agonist (500nM, 11914, Cayman Chemical, MI), and Wnt‐C59 (250nM, 5148, Tocris Bioscience, MN). On day 4, the cells were placed in accelerated spinal floorplate media for 24 h, consisting of Essential 6 Medium supplemented with retinoic acid (100 nM, R2625‐50MG, Sigma–Aldrich, MA) and DAPT (10 µM, 2634, Tocris Bioscience, MN). The iPSC‐derived sNPCs were then detached from the culture plate using Accutase (A6964, Sigma–Aldrich, MA) and resuspended in N2B27 media for printing. N2B27 was composed of Dulbecco's Modified Eagle Medium/F‐12 (11039‐047, Thermo Fisher Scientific, MA), 1 × CTS N‐2 (A13707‐01, Thermo Fisher Scientific, MA), 1 × Penicillin‐Streptomycin (15140‐122, Thermo Fisher Scientific, MA), and 1 × B‐27 (17504‐044, Thermo Fisher Scientific, MA).

### 5.1 Printing of Cell‐Laden Scaffolds

The cell‐laden scaffolds were generated via a custom‐built 3D printer equipped with multiple extrusion heads. The locations of these extrusion heads were controlled by a three‐axis Cartesian robot system (F5200N, Fisnar, WI). Multiple materials were extruded by a pneumatic dispensing system (Ultimus V, Nordson EFD, OH). 3D scaffolds with microscale channels were designed based on the size of the rat spinal cord for transplantation. The scaffolds were printed with silicone (Loctite SI 595, Henkel, OH) using a 150 µm diameter dispensing tip (7018424, Nordson EFD, OH) under optimized printing conditions (pressure: ≈140 psi, line speed: 0.8 mm s^−1^, and temperature: ≈21 °C) on a glass substrate (microscope slides, Premiere, China). They were cured after printing under ambient conditions for at least 5 h. A cell‐laden ink was prepared by mixing sNPCs (≈10^7^ cells mL^−1^) with 50% N2B27 neural media, 20 ng mL^−1^ NT‐3 (450‐03, PeproTech, NJ), 20 ng mL^−1^ brain‐derived neurotrophic factor (450‐02, PeproTech, NJ), 20 ng mL^−1^ glial cell‐derived neurotrophic factor (450‐10, PeproTech, NJ), and 50% Matrigel (354230, Corning, NY). Matrigel is liquid at low temperatures (< 4 °C) and solidifies at biological temperatures (> 10 °C). Matrigel consists of laminin, collagen IV, heparan sulfate proteoglycans, entactin/nidogen, and abundant growth factors. Thus, it aids in the long‐term survival and axonal growth of differentiated cells within the scaffolds, enabling the study of cellular behavior. The degradation of Matrigel occurs through the secretion of proteolytic enzymes produced by the cells themselves. The ink was printed into the microscale channels using a 100 µm diameter dispensing tip (7018462, Nordson EFD, OH) under optimized printing conditions (pressure: ≈0.7 psi, point dispense time: 0.3 s, and temperature: ≈4 °C). The tip temperature was maintained at ≈4 °C via a cooling system to maintain Matrigel in a liquid state for printability. The adhesion of the ink onto the scaffold channels was ensured by waiting 10 min after printing. The cell‐laden scaffolds for transplantation were specially designed with sacrificial layers to separate them from the substrates. Prior to printing the silicone scaffolds, the sacrificial layers were printed with 40% w/v Pluronic F‐127 using a 200 µm diameter dispensing tip (7018417, Nordson EFD, OH) under optimized conditions (pressure: ≈10 psi, line speed: 1 mm s^−1^, and temperature: ≈21 °C). The layers partially covered the bottom of the scaffolds and were dissolved during in vitro culture. G‐code commands were used to execute all print paths. The bioprinted cells were later fed with N2B27 supplemented with 20 ng mL^−1^ glial cell‐derived neurotrophic factor (450‐10, PeproTech, NJ), 20 ng mL^−1^ brain‐derived neurotrophic factor (450‐02, PeproTech, NJ), and 20 ng mL^−1^ NT‐3 (450‐03, PeproTech, NJ) as per Joung et al.^[^ ^30^ ^]^ The media was changed every 3 days.

### Tissue Clearing and IHC of 3D‐Printed Cell‐Laden Scaffolds

The tissue clearing technique was used to prepare the 3D tissue constructs within the scaffolds for volumetric imaging. It was performed on the 3D‐printed sNPC‐laden scaffolds (n = 3 scaffolds/group) at 15, 30, 40, 140, 170, and 365 days after printing, utilizing the SHIELD/SWITCH protocol developed by the Chung laboratory.^[^ ^51^ ^]^ Briefly, the samples were fixed and incubated with the SHIELD OFF solution (LifeCanvas Technologies, MA) at 4 °C with shaking for at least 2 days. Next, the samples were incubated with the SHIELD ON solution (LifeCanvas Technologies, MA) at 37 °C with shaking for 1 day. The samples were then placed in the Passive Clearing Buffer (LifeCanvas Technologies, MA) at 37 °C while shaking until they were cleared. 5 progressive rounds of phosphate‐buffered saline with Tween 20 (PBST) exchange were used to remove the Passive Clearing Buffer. The samples were then incubated in the SWITCH OFF solution with the following primary antibodies while shaking for at least 3 days: anti‐SMI312 (1:200, 837904, BioLegend, CA) for neurons and axons, anti‐Evx1 (1:250, ab220665, Abcam, Cambridge, UK) for V0 interneurons, anti‐ FOXP2 (1:250, ab16046, Abcam, Cambridge, UK) for V1 interneurons, anti‐Chx10 (1:250, ab9016, Abcam, Cambridge, UK) for V2a interneurons, anti‐MAP2 (1:500, ab5392, Abcam, Cambridge, UK) for mature neurons and dendrites, anti‐APC (1:100, ab15270, Abcam, Cambridge, UK) for mature oligodendrocytes, and anti‐GFAP (1:500, Z0334, Agilent Dako, CA) for astrocytes. Next, the samples were placed in the SWITCH ON solution for 1 day and then washed 5 times with PBST. Subsequently, they underwent the SWITCH OFF/ON procedure for the secondary antibodies, followed by 5 washes with PBST. The samples were then optically cleared using EasyIndex (LifeCanvas Technologies, MA) for imaging. Images were acquired using either a Nikon A1R FLIM confocal microscope or an RS‐G4 ribbon scanning confocal microscope. The percentage of cells expressing various markers at different time points was quantified relative to DAPI (Thermo Fisher Scientific, MA) and subsequently averaged.

### RNA Sequencing

Samples for RNA‐seq included 2D sNPC cultures and 3D sNPC‐laden scaffolds. These sNPCs were differentiated from the same batch of iPSCs as previously described. By the fifth day of differentiation, sNPCs were either 3D bioprinted as previously described or 2D sNPC cultures were plated on Matrigel for comparison (Corning, NY). At 25 days post‐differentiation from iPCS, the total RNA was extracted from the two sample groups for RNA‐seq. Total RNA samples were quantified using the RiboGreen fluorometric assay (Quant‐iT RiboGreen RNA Assay Kit, Thermo Fisher Scientific, MA), and RNA integrity was confirmed with capillary electrophoresis. The facility was provided by the University of Minnesota Genomics Core (UMGC). The purity of the RNA was assessed using a Nanodrop spectrophotometer (Thermo Fisher Scientific, MA) and a 2100 Bioanalyzer system (Agilent Technologies, CA). The RNA integrity number ranged from 9.1 to 10. Total deoxyribonucleic acid (DNA) contamination was quantified using the PicoGreen fluorometric assay (Quant‐iT PicoGreen dsDNA Assay Kit, Thermo Fisher Scientific, MA). Libraries were generated by UMGC using 250 ng of total RNA. The reverse transcribed mRNA in each sample, using random primers, resulted in paired‐end cDNA libraries, which were subsequently sequenced with a HiSeq 2500 system (Illumina, CA). The samples were further processed as per Lavoie et al.^[^ ^6^ ^]^ Paired‐end reads of 50 base pairs were conducted with 20 million reads per sample, distributed across four lanes to enhance coverage and error correction. Later, FASTQ files for each sample were combined, and the raw sequences were analyzed using a customized pipeline developed and maintained by the Minnesota Supercomputing Institute (https://bitbucket.org/jgarbe/gopher‐pipelines/wiki/rnaseq‐pipeline).^[^ ^52^ ^]^

Each FASTQ file underwent quality control with FastQC (v.0.11.5) both before and after trimming with Trimmomatic (v.0.33). The trimmed sequences were then aligned using HISAT2 (v2.02). Transcript abundance and differential gene expression were estimated using Cufflinks (v2.2.1). The reads were normalized and log‐transformed using edgeR. Heat maps were generated with the log‐transformed values using the heatmap package. Hierarchical clustering was performed according to Lavoie et al.,^[^ ^6^ ^]^ using the Euclidean distances and the average linkage clustering method, and was visualized using ggplot2. Trimmed sequences were aligned using the Homo sapiens (GRCh38) reference genome via HISAT2 (v2.02).

### Electrophysiology

Whole‐cell patch‐clamp recordings were made in current clamp mode using a MultiClamp 700B microelectrode amplifier (Molecular Devices, CA) to assess intrinsic membrane properties and action potential generation. Cells were visually targeted under differential contrast microscopy. Neurons were considered viable if they exhibited resting potential below −50 mV, leak conductance below 100 pA, and multi‐exponential voltage changes in response to injected current. To assess the functional electrophysiological properties of neurons, 10 cells from each group (either 2D cell cultures or 3D‐printed cell‐laden scaffolds) were analyzed. At room temperature, artificial cerebrospinal fluid containing 119 mM NaCl, 2.5 mM KCl, 1.0 mM NaH_2_PO_4_, 1.3 mM MgSO_4_, 2.5 mM CaCl_2_, 26.2 mM NaHCO_3_, and 11 mM glucose, saturated with 95% O_2_/5% CO_2_ (pH 7.4), was continuously perfused through the cultures. Picrotoxin 50 µM (Sigma–Aldrich, MA) was added to all electrophysiological recordings to block GABAergic neurotransmission. Pipettes (2.8–3.1 MΩ tip resistance) were pulled from capillary glass (Sutter Instrument, CA) and filled with an internal solution containing 122.0 mM K‐gluconate, 20 mM HEPES, 0.4 mM EGTA, and 2.8 mM NaCl.

### In Vivo Study Design

Athymic nude (ATN) rats underwent transection SCI. Female rats were chosen for this study because bladder expression is required during the acute period following injury. Male rats were more challenging to express, and based on the previous experience, many of them developed life‐threatening hematuria. ATN rats, which were athymic and T cell‐deficient, were utilized in the study to eliminate the need for immunosuppression for human cell transplant survival. Based on the previous experience, the weight of the ATN rats was standardized to a range of 230–250 g, and they were ≈10 weeks old at the time of injury.^[^ ^26^ ^]^ The smallest number of rats necessary to responsibly identify statistical significance was selected. According to the power analysis by Charan and Kantharia^[^ ^53^ ^]^ and the previous study,^[^ ^26^ ^]^ an estimated sample size of 15 rats (5 rats per group) was determined for the functional analysis. The effect size (1.0) for the BBB locomotor rating scale was used with 90% power in injured rats. The sample size was adjusted for an expected attrition or mortality rate of up to 10% due to the uncertainty of the tolerance of the rats to the injury and scaffolds. Therefore, a total of 18 ATN rats were subjected to transection injury and randomly assigned to one of three groups. During the study, 2 of these rats died from surgical complications. The first group received only the transection injury (n = 6), the second group received two empty scaffolds (n = 5), and the third group received two spinal cord organoid scaffolds (n = 5). The two scaffolds were aligned as previously described. Behavioral recovery was examined weekly for 12 weeks, and MEPs were recorded in all groups at the end of the study. Subsequently, tissue samples from sacrificed rats were analyzed for cell survival and differentiation patterns of the 3D bioprinted cells using standard IHC techniques. Each rat was considered an experimental unit, and the experiments were conducted in a blinded fashion.

### Surgical Procedures

The adult female ATN rats, ranging from 230 to 250 g, received a thoracic 8/9 (T8/9) transection injury according to the guidelines of the University of Minnesota Institutional Animal Care and Use Committee (Protocol No. 2304–41304A). At the T8/T9 vertebral level, laminectomies were performed on anesthetized rats. The spinal cord was then exposed and completely transected. Rats immediately received transplantation of the 3D bioprinted spinal cord organoid scaffolds, which were obtained by culturing the 3D‐printed sNPC‐laden scaffolds in vitro for 40 days after printing. After surgery, rats were subcutaneously injected with ceftiofur (1–20 mg kg^−1^) to prevent infection.

### Functional Studies

The BBB locomotor rating scale^[^ ^54^ ^]^ was used to evaluate behavioral performance by two blinded examiners. The BBB test was performed weekly for all groups over a period of 12 weeks.

### Motor Evoked Potentials

MEPs were conducted in all groups to examine spinal motor conduction, as described by Zhang et al.^[^ ^55^ ^]^ Briefly, a Magstim 200^2^ magnetic stimulator (Magstim, Whitland, UK), operating at a stimulating frequency of 0.25 Hz with a maximum voltage of 2.80 kV, was used to stimulate the motor cortex in all animals 12 weeks after surgery. An LR10 electrophysiological recording system (Tucker–Davis Technologies, FL) was used to record the waveform. 12 mm long monopolar needles were inserted into the gastrocnemius muscle as the recording electrode and into the distal tendon as the reference electrode. The interelectrode impedances were kept below 10 kΩ. The recording sampling rate was 6 kHz for all animals.

### Harvest of Tissue and Scaffold

The scaffolds and tissue samples were harvested at the end of 12 weeks post‐transplantation to examine cell survival, cell differentiation, and integration into the host spinal cord. The anesthetized rats were transcardially perfused with 4% paraformaldehyde (PFA) in 0.1 M PBS at pH 7.4. The harvested spinal cords were fixed overnight in 4% PFA, then immersed in 30% w/v sucrose, and subsequently washed with PBS. Some scaffold samples were subjected to the SHIELD/SWITCH tissue clearing assay (n = 3 rats/group), as detailed in Section 5.4. Some tissue samples were processed for cryosectioning (n = 3 rats/group) to enable IHC. These samples were collected from both sides of the injury site and embedded in a Tissue‐Tek OCT compound (Sakura Finetek, CA). They were cryosectioned in the transverse plane at 20 µm intervals with a Leica CM3050 S cryostat and then mounted onto positively charged slides.

### IHC of Transplanted Scaffolds

Transplanted scaffolds, processed using the SHIELD/SWITCH tissue clearing protocol, were labeled with SC121 and NF200 primary antibodies.^[^ ^6^ ^]^ Subsequently, the samples were treated with secondary antibodies and matched for refractive index using EasyIndex for imaging. Detailed procedures are described in Section 5.4.

### IHC of Spinal Cord Tissue Sections

The spinal cord tissue sections were labeled with the human cytoplasmic marker SC121 (1:250, Y40410, Takara Bio, CA) and its corresponding secondary antibody to identify the transplanted cells. This analysis employed 3 transverse sections of 20 µm thickness from T7 (≈5 mm rostral to the scaffold transplantation) and T10 (≈5 mm caudal to the scaffold transplantation). The average integrated optical density (fluorescence intensity per unit area)^[^ ^56^ ^]^ of SC121 positive projections was quantified using ImageJ software (Fiji v.1.45, NIH, MD).

Additionally, the percentage of transplanted surviving SC121 positive cells that co‐expressed or differentiated into APC or NF200 was quantified, as described by Patil et al.,^[^ ^26^, ^57^ ^]^ in the spinal cord sections of rats implanted with 3D‐printed organoid scaffolds. The spinal cord sections were also labeled with anti‐NF200 (1:250, ab8135, Abcam, Cambridge, UK) for intermediate filaments in neurons and anti‐APC (1:100, ab15270, Abcam, Cambridge, UK) for mature oligodendrocytes, along with their corresponding secondary antibodies. The SC121 positive cells that were double‐labeled with either APC or NF200 were counted. Both negative and positive controls were included to distinguish true biological signals from background noise. The cells were counterstained with DAPI (Thermo Fisher Scientific, MA).

### Statistical Analyses

Graph Pad Prism was used to conduct all the statistical analyses, and the data are presented as mean ± standard error of the mean as per the prior publications.^[^ ^26^, ^57^ ^]^ Statistical analysis for MEP was performed using one‐way analysis of variance (ANOVA) and with the Bonferroni post hoc test. Significance was set at ^*^ *p*< 0.05, ^#^ *p*< 0.01, and ^&^ *p* for non‐significance. A repeated measures ANOVA was used to statistically evaluate the BBB scores, with significance defined as ^*^ *p*< 0.05, ^#^ *p*< 0.01, ^c^ *p*< 0.001, and ^&^ *p* for non‐significance.

For the RNA‐seq data analysis, a two‐sided Student's *t*‐test was used to determine differential expression, with a global false discovery rate of 0.5. Transcript sizes and the count of aligned reads for each gene were normalized using cuffnorm, resulting in ‘Fragments per Kilobase of transcript per Million mapped reads’, representing relative expression levels.

## Conflict of Interest

The authors declare no conflict of interest.

## Supporting information



Supporting Information

Supplemental Movie 1

## Data Availability

All data required to evaluate the conclusions in the paper are included in the paper and/or the Supporting Information. Additional supporting data are available at the Data Repository for the University of Minnesota (DRUM) (https://doi.org/10.13020/ry5d‐ay17) and can be requested from the authors.
