# Supplementary material for: 3D‐Printed Scaffolds Promote Enhanced Spinal Organoid Formation for Use in Spinal Cord Injury
Source: Adv Healthc Mater. 2025 Jul 23;14(24):e04817. doi: 10.1002/adhm.202404817 (PMC12447032; doi:10.1002/adhm.202404817)
Supplement: Supplementary file 1 — Supporting Information [file ADHM-14-0-s001.docx]

Supporting Information

**3D Printed Scaffolds Promote Enhanced Spinal Organoid Formation for Use in Spinal Cord Injury**

*Guebum Han, Nicolas S. Lavoie, Nandadevi Patil, Olivia G. Korenfeld, Hyunjun Kim, Manuel Esguerra, Daeha Joung, Michael C. McAlpine, and Ann M. Parr**

G.H., N.S.L., and N.P. contributed equally to this work

* Corresponding author

Dr. G. Han, H. Kim, Prof. M. C. McAlpine

Department of Mechanical Engineering, University of Minnesota, Minneapolis, MN 55455, USA.

N. S. Lavoie, Dr. N. Patil, O. G. Korenfeld, Prof. A. M. Parr

Department of Neurosurgery, Stem Cell Institute, University of Minnesota, Minneapolis, MN 55455, USA.

E-mail: amparr@umn.edu

Dr. M. Esguerra

Department of Neuroscience, University of Minnesota, Minneapolis, MN 55455, USA.

Prof. D. Joung

Department of Physics, Virginia Commonwealth University, Richmond, VA 23284, USA.


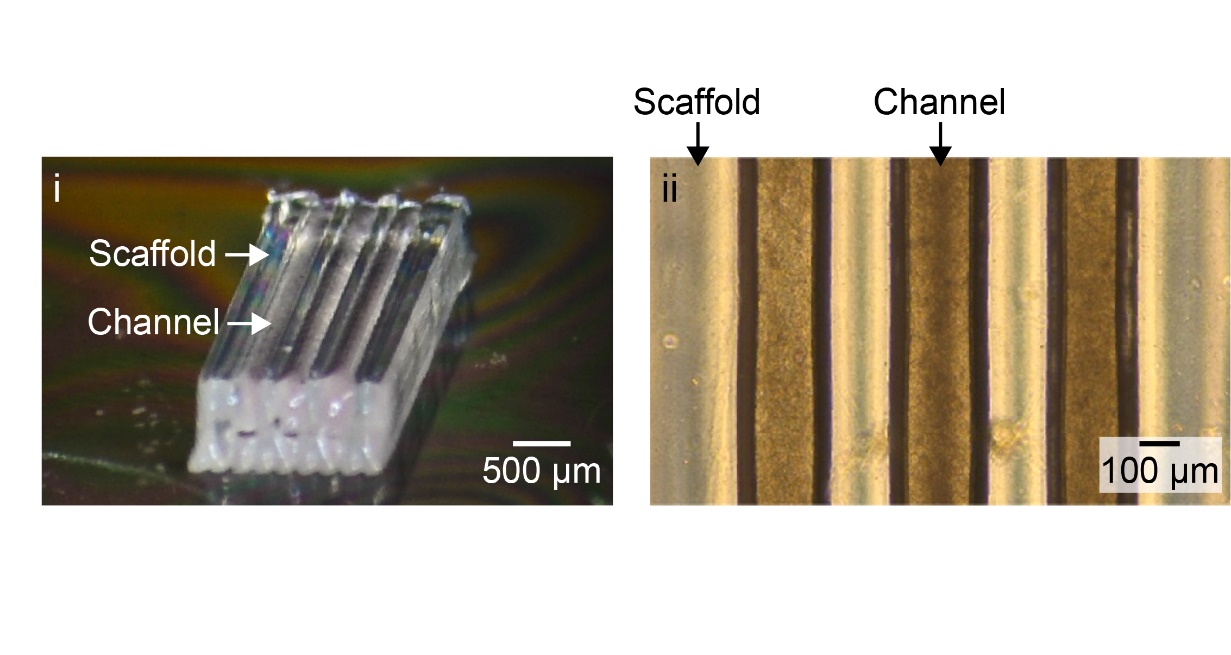


**Figure S1.** 3D printed spinal neural progenitor cell (sNPC)-based silicone scaffold. (i) The photograph shows a scaffold with a length of 5 mm. (ii) The photograph shows the scaffold containing three 200 µm wide channels, each filled with sNPCs. The image was acquired 2 weeks post-printing.


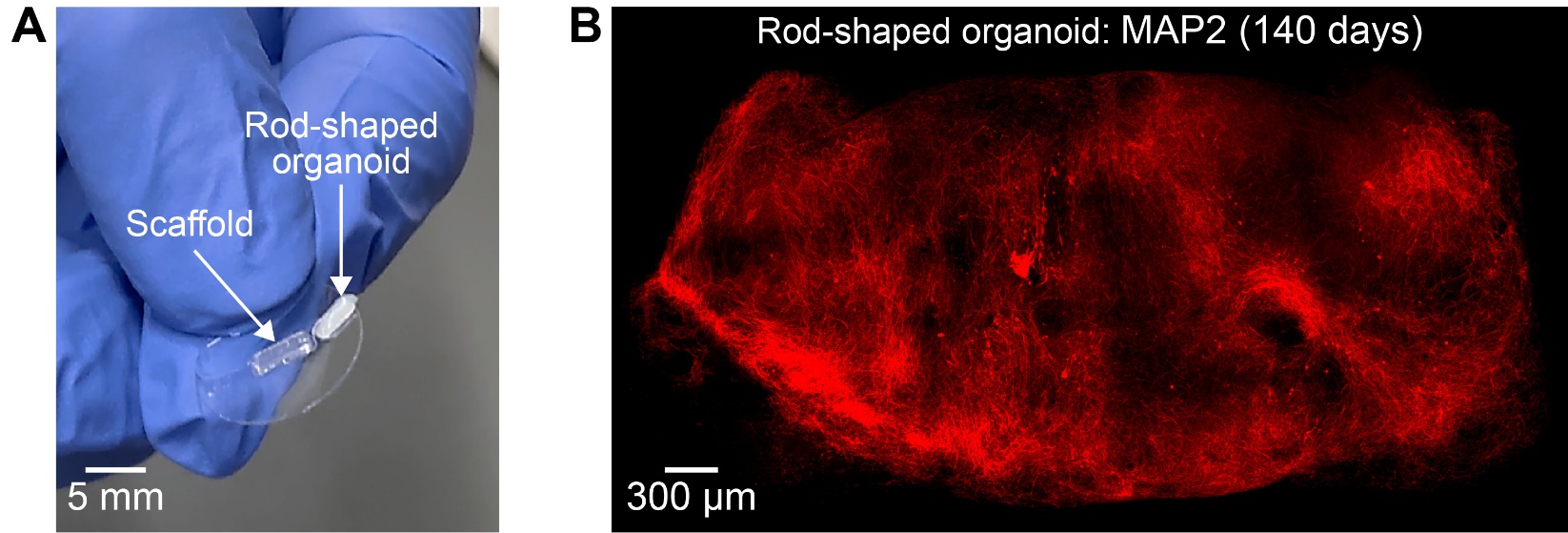


**Figure S2.** **A**. Image showing a 5 mm scaffold and a rod-shaped organoid. The rod-shaped organoid was detached from the scaffold for imaging. **B**. Expression of MAP2 showing neurons that grew along the 5 mm scaffold at 140 days.


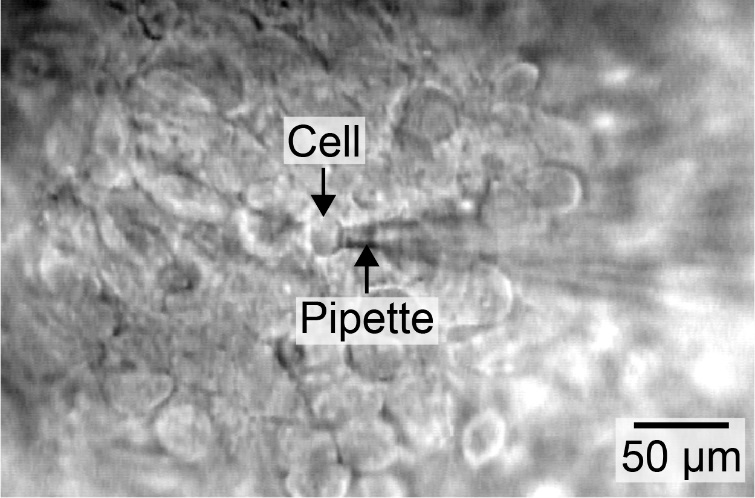


**Figure S3.** Cells and a pipette under differential contrast microscopy for whole-cell patch-clamp recordings.


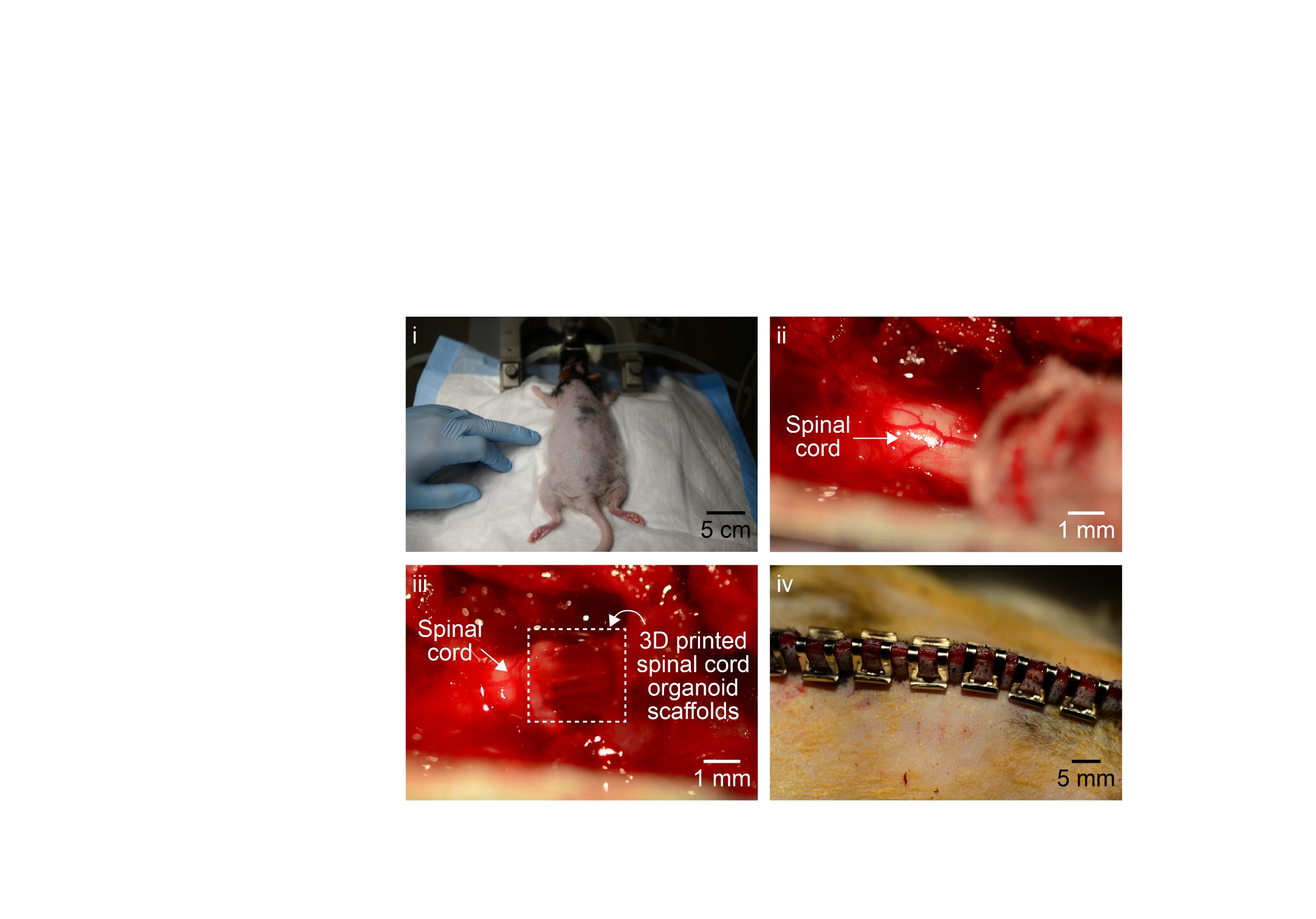


**Figure S4.** Transplantation process of 3D printed spinal cord organoid scaffolds: (i) anesthesia, (ii) exposure of the spinal cord of the rat, (iii) transplantation of the 3D printed spinal cord organoid scaffolds into the 1.8 mm gap in the spinal cord created by the transection injury, and (iv) closure of the surgical site.


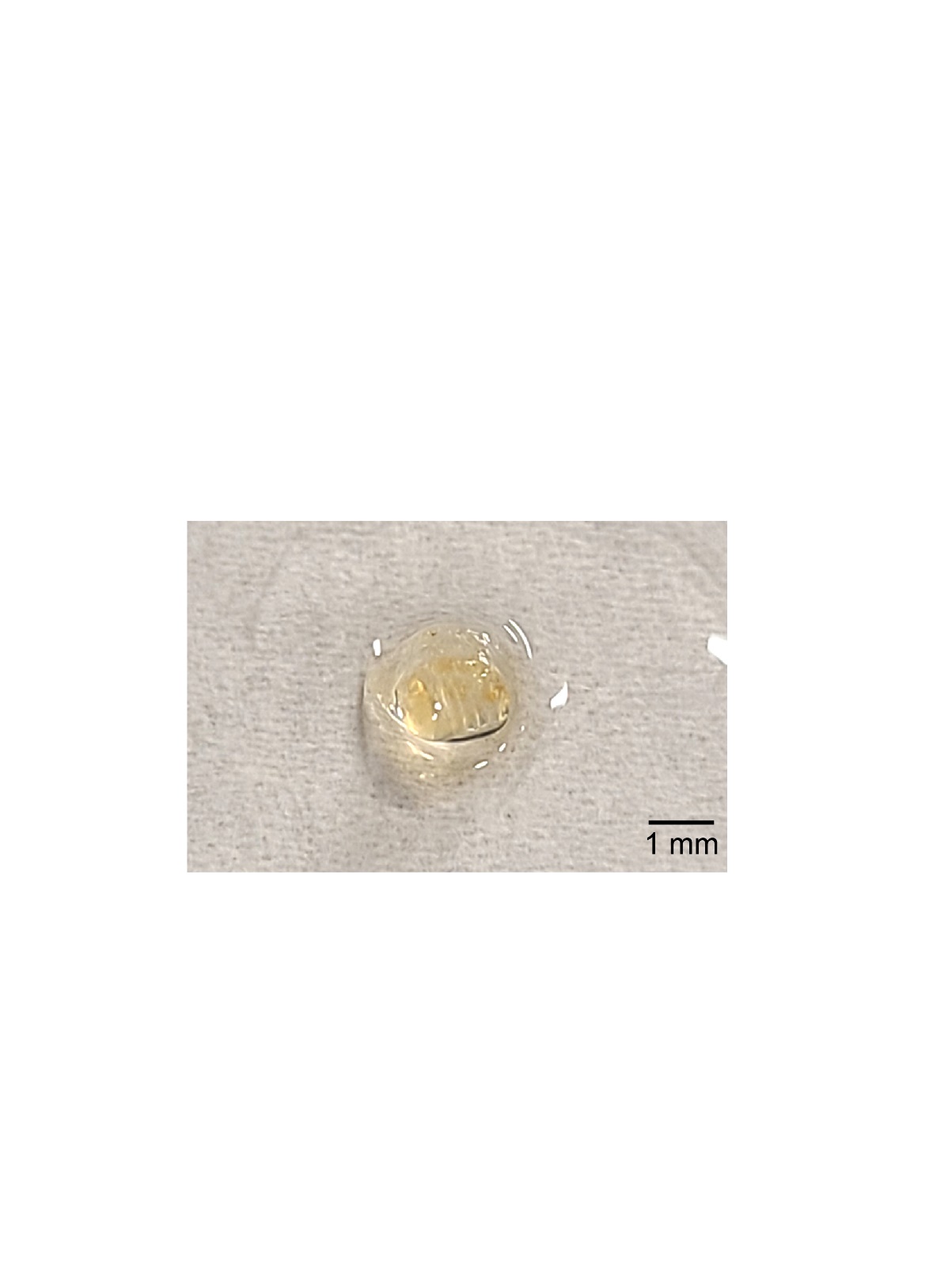


**Figure S5.** Tissue clearing of a 3D printed spinal cord scaffold and adjacent host tissue 12 weeks post-transplantation in a rat.


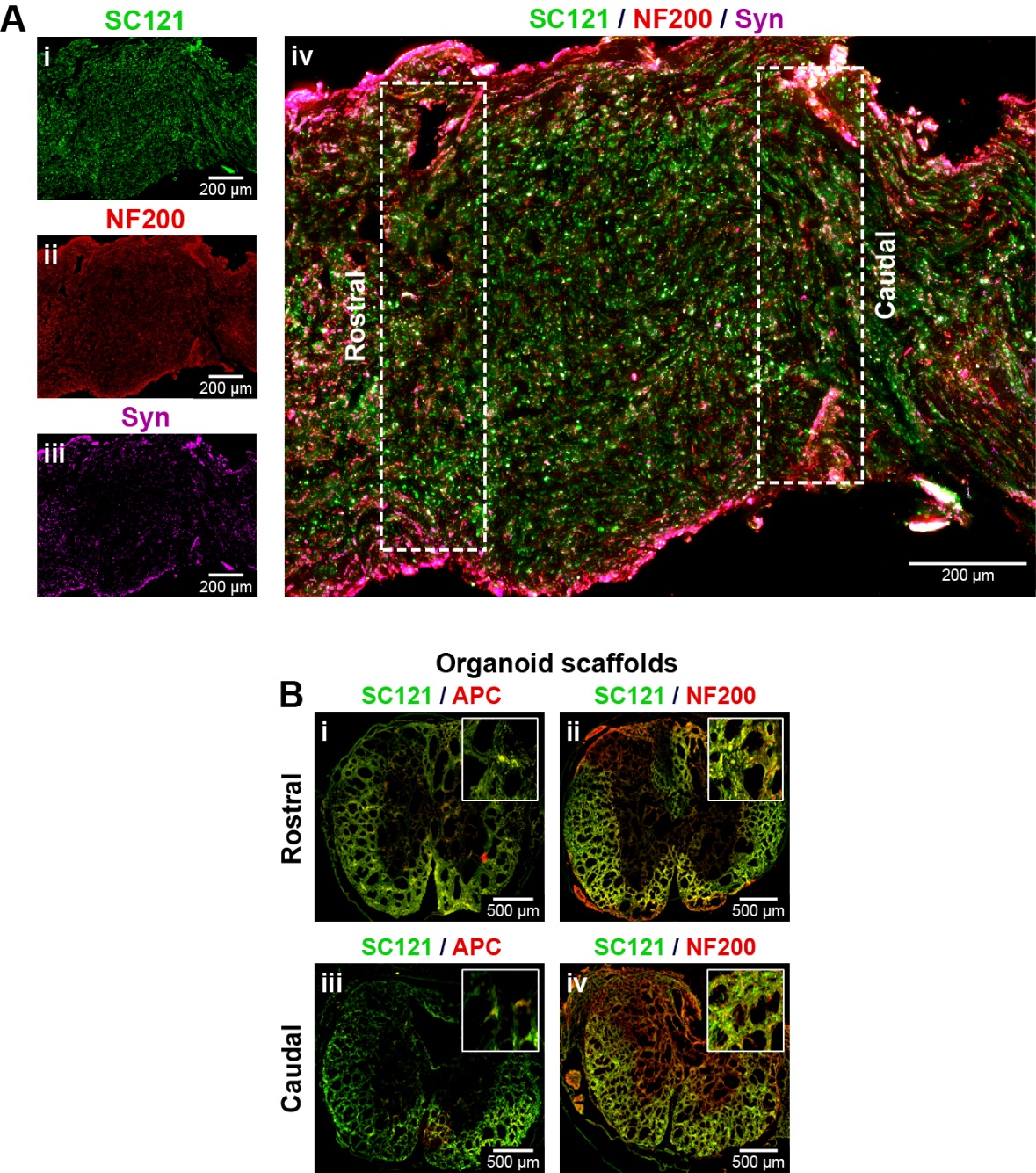


**Figure S6.** **A**. Axons regenerated across the graft–host interface. (i) SC121 (green), (ii) NF200 (red), (iii) Synaptophysin (Syn, magenta), and (iv) co-localization of SC121, NF200, and Syn in the scaffold 12 weeks post-transplantation. This IHC result was obtained from a thin 2D tissue section after removal of the scaffold implant. The implanted cells integrated near the bridging areas and regenerated tissue across the interfaces at both ends of the scaffold implant, as highlighted by the dotted boxes. **B**. Expression of SC121 co-labelled either with APC or NF200 in spinal cord sections from the organoid scaffold group. (i and ii) Rostral to the organoid scaffold implants. (iii and iv) Caudal to the organoid scaffold implants.

Movie S1. 3D Printed Spinal Cord Scaffolds
